# A Well-Controlled Experimental System to Study Interactions of Cytotoxic T Lymphocytes with Tumor Cells

**DOI:** 10.3389/fimmu.2016.00326

**Published:** 2016-08-30

**Authors:** Natalie J. Neubert, Charlotte Soneson, David Barras, Petra Baumgaertner, Donata Rimoldi, Mauro Delorenzi, Silvia A. Fuertes Marraco, Daniel E. Speiser

**Affiliations:** ^1^Department of Oncology, Ludwig Center for Cancer Research, University of Lausanne, Epalinges, Switzerland; ^2^Bioinformatics Core Facility, SIB Swiss Institute of Bioinformatics, Lausanne, Switzerland

**Keywords:** melanoma, CD8+ T cell, co-culture, antigen-specific, NanoString

## Abstract

While T cell-based immunotherapies are steadily improving, there are still many patients who progress, despite T cell-infiltrated tumors. Emerging evidence suggests that T cells themselves may provoke immune escape of cancer cells. Here, we describe a well-controlled co-culture system for studying the dynamic T cell – cancer cell interplay, using human melanoma as a model. We explain starting material, controls, and culture parameters to establish reproducible and comparable cultures with highly heterogeneous tumor cells. Low passage melanoma cell lines and melanoma-specific CD8+ T cell clones generated from patient blood were cultured together for up to 3 days. Living melanoma cells were isolated from the co-culture system by fluorescence-activated cell sorting. We demonstrate that the characterization of isolated melanoma cells is feasible using flow cytometry for protein expression analysis as well as an Agilent whole human genome microarray and the NanoString technology for differential gene expression analysis. In addition, we identify five genes (*ALG12, GUSB, RPLP0, KRBA2*, and *ADAT2*) that are stably expressed in melanoma cells independent of the presence of T cells or the T cell-derived cytokines IFNγ and TNFα. These genes are essential for correct normalization of gene expression data by NanoString. Further to the characterization of melanoma cells after exposure to CTLs, this experimental system might be suitable to answer a series of questions, including how the affinity of CTLs for their target antigen influences the melanoma cell response and whether CTL-induced gene expression changes in melanoma cells are reversible. Taken together, our human T cell – melanoma cell culture system is well suited to characterize immune-related mechanisms in cancer cells.

## Introduction

Recently, there have been major breakthroughs in treating cancer patients using T cell-based immunotherapy (1, 2), which are promising in cancer types that are immunogenic and patients capable to mount an immune response. Prototypes of immunogenic tumors include melanoma, non-small cell lung cancer, prostate cancer, and ovarian cancer. Beyond, the list of malignancies responding to immunotherapy is rapidly growing (3).

The most successful T cell-based therapies for solid cancers are adoptive T cell transfer with autologous tumor-infiltrating T cells, and inhibitory receptor blockade (checkpoint blockade) (3). At the center of these therapies are cytotoxic CD8+ T lymphocytes (CTLs) that can eliminate tumor cells. While such therapies prolong patient survival, there is still an important fraction of patients where cancers escape from immune attack. Experiments in mice have shown that intratumoral CTLs can trigger tumor cells to upregulate inhibitory receptor ligands (e.g. PDL1) and the immune suppressive enzyme IDO (4, 5), suggesting that CTLs themselves might contribute to provoke escape from immunotherapy. The large majority of publications describe long-term escape variants of tumor cells (6, 7), mouse models (4, 8), and data from human bulk tumors (9, 10), which are suboptimal for individualized and broad characterization of the immediate interplay between specific cell types of the tumor microenvironment.

Melanomas develop from melanocytes, pigmented cells mostly found in the skin but also in the eye and inner ear. Melanoma cells express melanoma-specific antigens, such as the well-characterized melanoma differentiation antigens MelanA (MART-1), gp100 (pmel) and tyrosinase (11–13) that can be recognized by CTLs via their T cell receptors (TCRs). Many melanoma-specific human CTLs and their TCRs have been studied in depth (14–16). Thus, human melanomas are well suited for studying CTL–tumor cell interactions.

To date, an *in vitro* system to comprehensively study the immediate interactions of CTLs and tumor cells is missing. We believe that short-term co-cultures of melanoma cells with CTLs can be useful for studying their dynamic interplay. The challenge of setting up a human *in vitro* co-culture system consists in the choice of appropriate cellular material and experimental parameters that lead to reproducible results despite that the cells are from highly heterogeneous melanoma patients. Nevertheless, we succeeded in establishing suitable methods, and herewith describe the starting material, controls, culture parameters, and readouts.

## Materials and Methods

### Cells and Cell Culture

All cell lines and clones were established at Ludwig Cancer Research, Department of Oncology, University of Lausanne. Patients consented based on approval of this work obtained from the local ethics committee. Melanoma cell lines were established from metastatic surgery specimens from melanoma patients (Table 1). Melanoma cells were cultured in RPMI 1640 – GlutaMAX^TM^-I, complemented with 10% heat-inactivated FCS (PAA), 1.1 μM arginine (Sigma Aldrich), 0.48 μM asparagine (Sigma Aldrich), 11.25 μM glutamine (Gibco), 10 mM Hepes (Gibco), and 100 U/ml of penicillin/streptomycin (Gibco). Where indicated, the medium was supplemented with IFNγ (222 U/ml; Peprotech), TNFα (50 ng/ml; Peprotech) or both TNFα and IFNγ (10 ng/ml and 222 U/ml). CD8+ T cell clones were established from antigen-specific CD8+ T cells isolated from PBMCs of melanoma patients or healthy donors and maintained as previously described (17, 18) (Table 2). CD8+ T cell clones were cultured in RPMI 1640 – GlutaMAX^TM^-I, supplemented with 100 U/ml of penicillin/streptomycin, 2 mM l-glutamine, 1 mM non-essential amino acids, 1% Na pyruvate, 0.1 mg/ml Kanamycin (all Gibco), 5 × 10^−5^ 2β-mercaptoethanol (Sigma) and 8% human serum. Human serum was prepared in house from serum of 30 male A+ donors obtained from the CRS Interregional Transfusion Center Bern. Serum was subject to tests of proliferation, mixed lymphocyte culture, and mycoplasma PCR.

**Table 1:** **The melanoma cell lines were derived from metastases or tumor-infiltrated lymph nodes of HLA-A2 positive melanoma patients**.

Cell line	Patient	Gender	Age at surgery (years)	Source of cell line	HLA-A
Me275	LAU50	Male	65	Lymph node metastasis	*02:01, *23:01
Me290	LAU203	Female	65	Lymph node metastasis	1, *02:01
T1015A	LAU1015	Male	77	Non-lymphoid metastasis	*02:09, 32(19)
T1185B	LAU1185	Female	60	Non-lymphoid metastasis	*02:01

*For the HLA-typing, numbers preceded by an asterisk were determined by molecular biology and the remaining ones by serology. The first one to two digit(s) stand for the “allele group”, whereas the four digits stand for the HLA protein*.

**Table 2:** **Antigen specificity of CTL clones derived from melanoma patients or health donors**.

Clone	Patient	Specificity
Clone 1	LAU1185 (melanoma patient)	A2/MelanA; EAAGIGILTV (melanoma)
Clone 121	LAU1015 (melanoma patient)	A2/MelanA; EAAGIGILTV (melanoma)
Clone N8	LAU5048 (healthy donor)	A2/NS4b; LLWNGPMAV (Yellow-fever virus)
Clone 12	BCL7 (healthy donor)	A2/BMLF1; GLCTLVAML (Epstein–Barr virus)

All cell lines and T cells were routinely controlled to be mycoplasma-free using PCR. The precise details and setup of the co-culture system are described throughout the results section.

### Fluorescence-Activated Cell Sorting

Before seeding, melanoma cells were labeled with 1 μM CFSE (Cell trace CFSE cell proliferation kit, Molecular Probes) and CTLs were labeled with 1 μM Violet tracker (Cell trace Violet Cell Proliferation kit, Molecular Probes). For intracellular staining, cell cultures were treated with BrefeldinA (final concentration: 10 μg/ml, Sigma Aldrich) for the last 4 h of culture. After harvesting, cells were washed once with PBS, stained with LIVE/DEAD^®^ Fixable Aqua Dead Cell Stain Kit (Life Technologies) for 30 min and fixed at 4°C overnight in PBS containing 1% Formaldehyde, 2% Glucose, and 5 mM NaAzide. Then cells were washed once with fluorescence-activated cell sorting (FACS) buffer (PBS supplemented with 5 mM EDTA, 0.2% BSA, and 0.2% NaAzide), stained with cell surface antibodies for 20 min followed by permeabilization and intracellular staining with antibodies in 0.1% saponin-containing FACS buffer for 30 min. The following anti-human antibodies were used: MelanA (clone A103, produced and labeled in house), HLA-DR (clone LN3, eBioscience), HLA Class I (clone W6/32, Biolegend), CD8 (clone SK1, BD), IFNγ (clone B27, BD), and TNFα (clone MAb11, BD). The following matched isotype controls were used: mouse IgG1 (produced in house, batch 24.11.14), mouse IgG2b kappa (clone eBMG2b, eBioscience), mouse IgG2a kappa (clone MOPC-173, Biolegend), mouse IgG1 kappa (clone MPC-11, BD), and IgG1 kappa (clone MOPC-21, BD). PE-labeled A2-ELA-specific tetramers (No. 404, TC Metrix) were used to stain MelanA-specific CTLs. AnnexinV staining was performed using fresh AnnexinV-binding buffer (14 mM NaCl, 2.5 mM CaCl2, 10 mM Hepes) containing AnnexinV-FITC (BD Pharmingen). Where indicated, cells were stained with DAPI nucleic acid stain (Molecular Probes) at 150 ng/ml for FACS acquisition and 3 μg/ml for FACS sorting. Cells were acquired using the Gallios flow cytometer (Beckman Coulter, 3-laser configuration) and data were analyzed using FlowJo (Tree Star Inc., v9.7.7). FACS sorting was performed with an Astrios cytometer (Beckman Coulter).

### Chromium Release Assay

To evaluate the antigen-specific killing capacity of CTLs, a chromium (^51^Cr) release assay was performed as described previously (17). In brief, melanoma cells were pulsed with ^51^Cr and cultured for 4 h at the indicated ratios with CD8+ T cell clones. Then, co-culture supernatant was transferred to LumaPlate™ 96-well plates (PerkinElmer) and its ^51^Cr content quantified with a Top Count NXT plate reader (PerkinElmer) as a measure of melanoma cell lysis.

### RNA Extraction

Cells were sorted directly into suspension media (PBS supplemented with 10% BSA and 0.05 M EDTA) and RNA was extracted using the Pico Pure™ RNA isolation kit (Arcturus) following the manufacturer’s Protocol B “RNA Extraction from Cell Pellets”. The optional freezing at −80°C directly after cell lysis and the optional on-column DNA digest were performed according to this protocol. The Qubit fluorometer (Life Technologies) was used to quantify the RNA and the 2100 Bioanalyzer (Agilent) served to determine the quality of the samples.

### Microarray

A total of 1000 viable (DAPI negative) melanoma cells were sorted into SuperAmp lysis buffer (Miltenyi Biotec). RNA was isolated and gene expression was measured using an Agilent Whole Human Genome 8 × 60K (V2) microarray as described by Fuertes Marraco et al. (19). The microarray data have been deposited in the NCBI Gene Expression Omnibus database with the accession number GEO: GSE79991.

### NanoString

RNA abundance was quantified with a custom-made set of probes by the iGE3 Genomics platform (University of Geneva) using the Prep Station and nCounter devices (NanoString Technologies) as previously described (20). The R software (version 3.2.2) and the NanoStringNorm package (version 1.1.19) were used for data analysis. First the average count of the negative controls was subtracted from the raw counts. Then the data were log2-transformed. “NA” replaced undefined values (i.e. the ones resulting from log transformation of zero count values). Afterwards, a shift based on the normalization genes was defined using simple regression with the mean as offset. When log fold-changes were computed, a value of 10 was added to all normalized counts, the counts were averaged per condition, fold-changes were computed (treated/untreated) and eventually log2-transformed. We added 10 to all counts before fold-change calculations to reduce the bias toward extremely high fold-change values when a gene had a very low expression level in the untreated condition.

## Results

### Characteristics of Melanoma Cell Lines and CD8+ T Cell Clones

For our co-cultures, we selected low passage human melanoma cell lines that had been generated from melanoma metastases or tumor-infiltrated lymph node specimens (Table 1). The cell lines had been passaged for less than 6 months at the time of the experiments. To allow antigen-specific CTL–melanoma cell interactions with MelanA-specific CTL clones, we chose melanoma cell lines expressing MelanA and HLA Class I as determined by flow cytometry (Figure S1A in Supplementary Material). All melanoma cell lines were derived from HLA-A2 positive patients (Table 1).

MelanA-specific CTLs had been previously cloned from patient blood (21). We selected CTLs specific for HLA-A2 and the MelanA_26–35_ decapeptide EAAGIGILTV that were capable of killing at least 50% of the tumor cells in a standard 4-h killing assay at a CTL:melanoma cell ratio of 10:1 (Table 2; Figures S1B and S2A in Supplementary Material). Killing assays with CTLs specific for non-melanoma antigens, such as epitopes from yellow fever virus (YFV) or Epstein–Barr virus (EBV), were used as negative controls to test if the observed CTL-induced reactions in tumor cells are dependent on antigen-specific interactions (Table 2). The killing was antigen-specific as MelanA-specific CTLs did not kill a MelanA-negative melanoma cell line (Figure S2B in Supplementary Material) and negative control CTLs did not kill MelanA-expressing melanoma cell lines (Figure S2C in Supplementary Material). We estimated that such MelanA-specific CTLs exert significant immune pressure and consequently provoke changes in the melanoma cells in co-cultures.

### Setup of Co-Culture

Melanoma cell lines are very heterogeneous in terms of growth speed, medium consumption (as determined by color of phenol-containing medium) and time needed for the cells to adhere in tissue culture plates. For the co-culture, we seeded melanoma cells 2–3 days in advance, and then allowed interaction with CTLs for 3 days. Therefore, to establish comparable growth conditions, we determined for each cell line the seeding density at which cells would not be confluent before 6 days in any of the culture conditions. Depending on the cell line, between 35,000 and 70,000 cells were seeded per 24-well in 2 ml. In addition, seeding densities were chosen based on only one medium change during the experiment (on the day that CTLs were added). This served to avoid removing any secreted factors that might participate in the co-culture.

To distinguish melanoma cells and CTLs by flow cytometry (FACS), melanoma cells were CFSE-labeled and CTLs violet tracker-labeled (Figure S3A in Supplementary Material). This was necessary, because during co-cultures, melanoma cells and CTLs change morphology, making it impossible to clearly separate the cell types based on forward and sideward scatter (Figure S3B in Supplementary Material).

**Figure 1:**
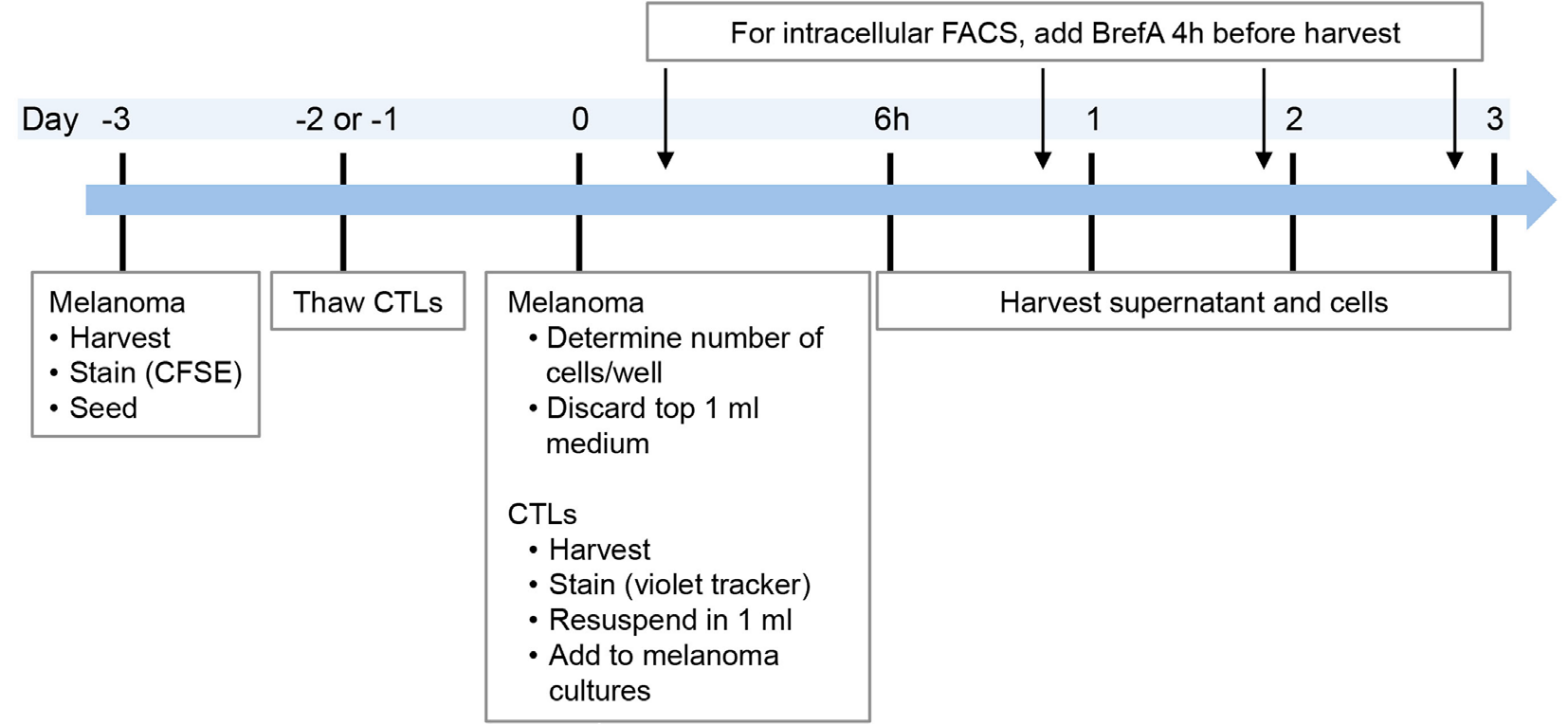
**Timeline of co-culture experiments**. In the case of intracellular FACS requiring addition of BrefeldinA, supernatant was taken before addition of BrefeldinA or from replicate cultures without BrefeldinA. Melanoma cell lines were thawed at least 1 week prior to the experiment.

The outline of the co-culture procedure was as follows: melanoma cell lines were seeded in advance (in general 3 days prior to the start of the co-culture) to allow them to adhere to the plastic (Figure 1), in 24-wells and 2 ml volume. On the day of co-culture start (day 0), 1 ml of medium was discarded and replaced with 1 ml of fresh medium containing the respective treatment, i.e., CTL clones, cytokines (IFNγ, TNFα), or no treatment.

To maintain CTL viability, 30 U/ml of IL2 was added at treatment start, which was done for all cultures. Presence of IL2 did not influence melanoma cells as we found similar protein expression with or without (data not shown). Depending on the condition of the melanoma cell lines, growth speed differed between experiments. Consequently, it was necessary to determine the melanoma cell number in two additional control wells on day 0 in order to calculate the number of CTLs required for the appropriate CTL:melanoma cell ratio (Figure 1).

### Ratio and Viability of CTLs and Melanoma Cells in Co-Culture

**Figure 2:**
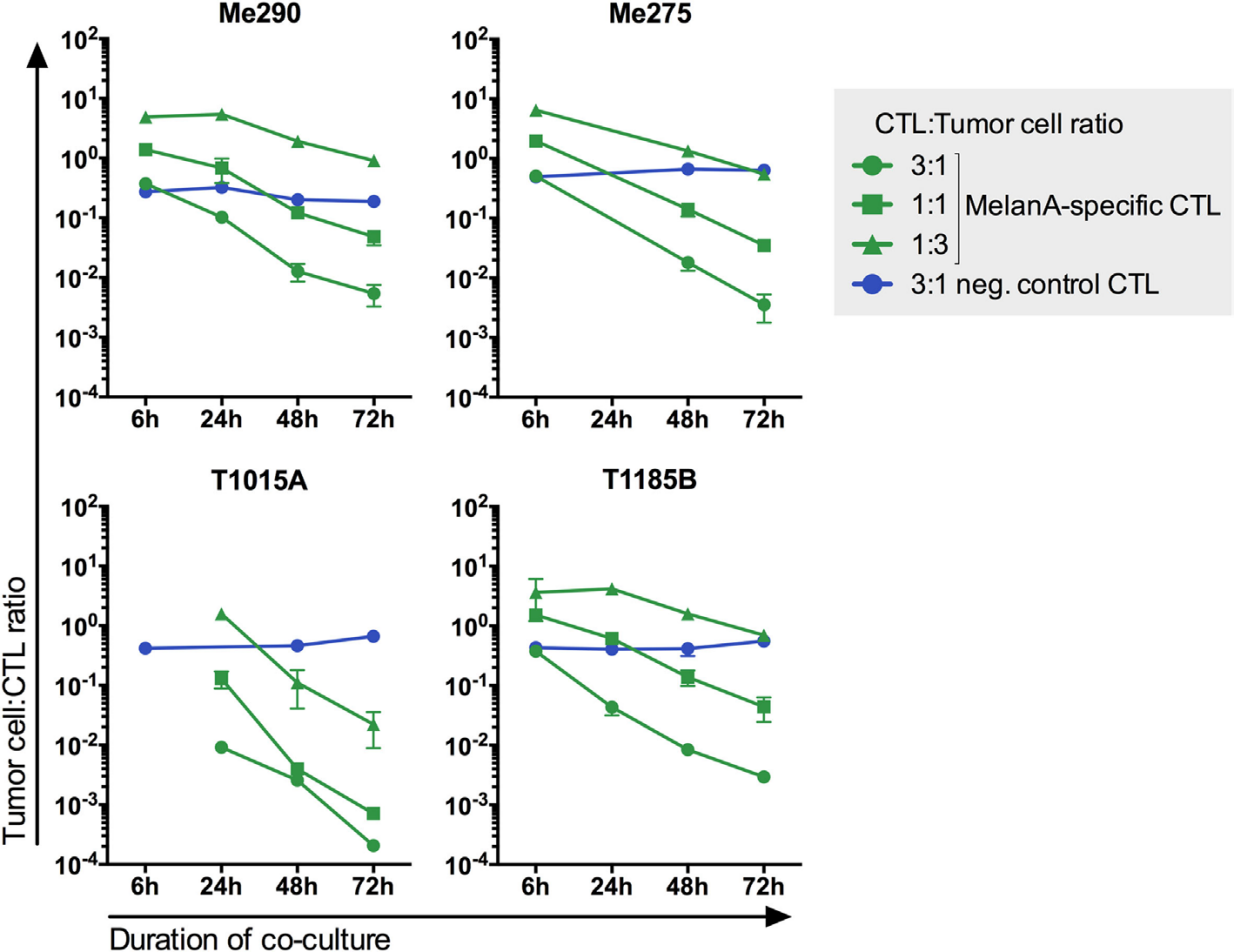
**CTL-mediated killing of melanoma cell lines in co-cultures is antigen-specific**. Three different MelanA-specific CTL:melanoma cell ratios are shown in green. Yellow-fever-virus- or Epstein–Barr-virus-specific CTLs did not kill the melanoma cells (blue). The number of living cells was measured by flow cytometry. *N* = 2, except for T1185B 1:3 with MelanA-specific CTLs and 3:1 with neg. control CTLs *N* = 4. Error bars indicate SD.

To simulate co-existence of tumor-infiltrating CTLs and melanoma cells, we assessed the CTL:melanoma cell ratios where both cell types can persist for up to 3 days. Six CTL:melanoma cell ratios were tested (10:1, 1:1, 1:3, 1:10, 1:30, and 1:100) (data not shown). The three ratios showing co-existence during the co-culture (3:1, 1:1, and 1:3) were chosen for further experiments. As expected, the melanoma cell:CTL ratio decreased during the 3-day co-culture, indicating tumor cell killing by CTLs and/or CTL proliferation (Figure 2; Figure S4 in Supplementary Material). MelanA-specific but not non-specific CTLs (YFV- or EBV-specific CTLs) killed the melanoma cell lines, demonstrating that the killing was antigen dependent. These observations are in accordance with the 4-h-killing assay shown in Figure S2 in Supplementary Material.

To harvest the maximum number of viable cells, we tested different harvesting methods. Melanoma cells are adherent, whereas CTLs are not adherent. In addition, melanoma cells are fragilized in presence of MelanA-specific CTLs or their cytokines IFNγ and TNFα. Thus, quick and gentle harvesting was necessary. Collecting the supernatant, followed by one wash with PBS, a short incubation with accutase and harvesting the detached cells yielded the highest number of viable melanoma cells (Figure S5 in Supplementary Material). All liquids, including supernatant and washes, were collected.

To optimize viability of cells and achieve high-quality data, we minimized intervention on the cells after harvest. Particularly for RNA-based analyses rapid processing was crucial for RNA quality. To achieve lysis of sorted cells for RNA extraction within 2 h from start of harvesting, no stainings were performed on harvested cells except from addition of DAPI for dead cell exclusion. This was possible, because melanoma cell lines and CTLs had been labeled with CFSE and violet tracker prior to co-culture. Furthermore, samples were harvested sequentially to avoid waiting times before sorting.

### CTL Function in Co-Culture

**Figure 3:**
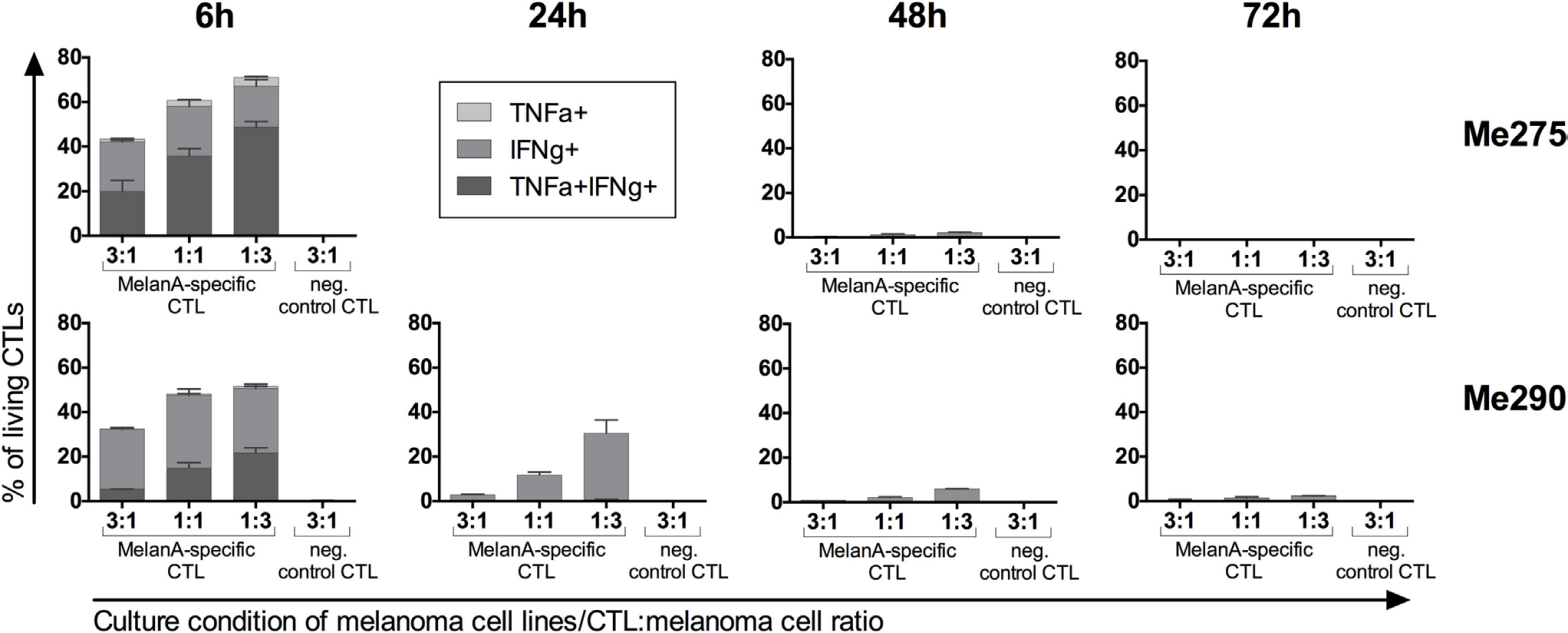
**Co-cultured antigen-specific CTLs produce cytokines**. MelanA-specific CTLs accumulate intracellular IFNγ and TNFα with strongest expression at 6 h of co-culture with the melanoma cell lines Me275 (top row) and Me290 (bottom row). Non-specific CTLs did not accumulate intracellular cytokines. Cytokine expression was measured by flow cytometry after intracellular staining of the co-cultured cells. *N* = 2, error bars indicate SD.

Upon activation and antigen recognition, CTLs can not only kill target cells but also secrete the cytokines IFNγ and TNFα. To evaluate the functionality of antigen-specific CTLs, we quantified IFNγ and TNFα protein expression. Vesicle transport was blocked with BrefeldinA during 4 h before harvesting the co-cultures to allow intracellular FACS analysis. Indeed, antigen-specific but not control CTLs accumulated IFNγ and TNFα during the co-culture (Figure 3). The cytokines accumulated primarily at 6 h. In the co-cultures with the lowest CTL-to-melanoma cell ratio (1:3), the percentages of cytokine-producing CTLs were highest. Furthermore, expression of IFNγ was more frequent than TNFα. Our observations are in accordance with the published literature indicating high cytokine production by CTLs when exposed to more tumor cells per CTL (22).

### Time-Point 48 h Is Optimal for Protein Expression Analysis

**Figure 4:**
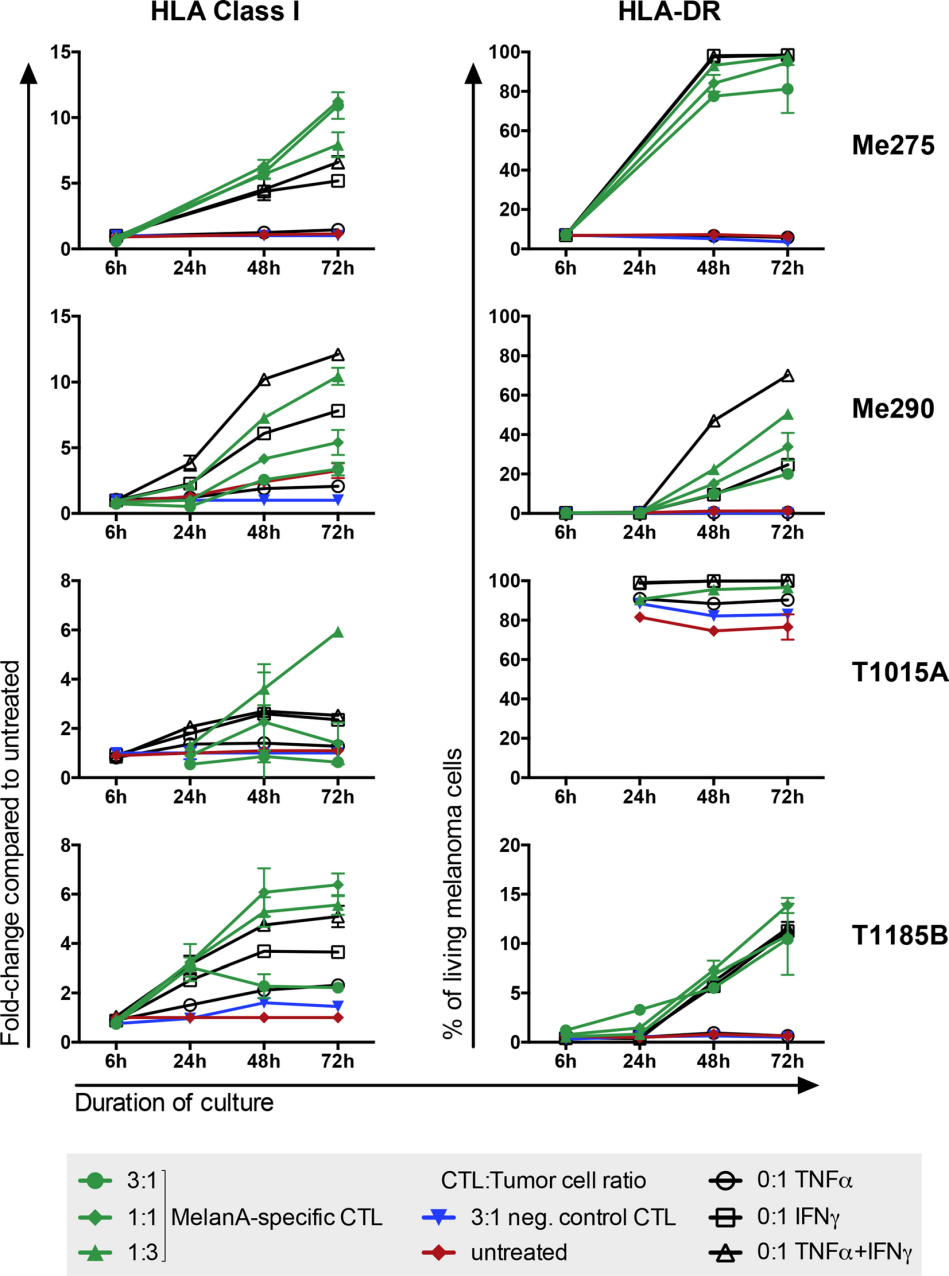
**Melanoma cells increase HLA Class I and HLA-DR expression upon exposure to CTLs**. Exposure of different melanoma cell lines to MelanA-specific CTLs (green) or CTL-derived cytokines (black) but not to non-specific CTLs (blue) provoked changes of protein expression on the melanoma cells. Untreated cells are shown in red. HLA Class I was calculated as fold-change compared to untreated cells. HLA-DR is presented as % positive of living cells. Measurements were performed by flow cytometry. *N* = 2, error bars indicate SD.

To test if the co-culture is a valid system to investigate CTL–tumor cell interactions, we analyzed two markers whose behavior is known to change upon exposure to IFNγ or CTLs: HLA-DR and HLA Class I (23, 24). In addition, with the help of these markers we wanted to determine the best time-point to analyze melanoma cell responses to CTLs. As expected, although all cell lines expressed HLA Class I when untreated, its expression further increased in all cell lines upon co-culture (Figure 4). HLA-DR expression also increased in three out of four co-cultures. The three melanoma cell lines that showed an increase in HLA-DR expression had no or very low HLA-DR expression when left untreated whereas in the fourth cell line, T1015A, HLA-DR was constitutively expressed. Taken together, HLA Class I and HLA-DR behaved as expected with increased expression upon exposure to CTLs or their cytokines, supporting that the co-culture system is a valid approach to investigate tumor cell reactions to CTLs. For protein expression analysis, the 1:1 CTL:melanoma cell ratio at a 48-h time-point was the most appropriate setup, because strong protein expression changes were measured and, in addition, sufficient numbers of living melanoma cells could be harvested for reliable protein expression analysis.

### Time-Point 24 h Is Suitable for mRNA Screen of Co-Cultured Melanoma Cells

Currently, most screening methods for exploratory analyses of factors relevant to a biological process rely on mRNA samples. Screenings at the protein level, such as mass spectrometry-based proteomics, are still subjected to technical obstacles, such as lack of reproducibility (25), and require large amounts of cellular material. Thus, in order to explore changes at the genome-wide level and to allow for the detection of unexpected candidates, we wanted to test if the co-culture system is suitable for measurement of gene expression by microarrays.

First, we performed quantitative PCR (qPCR) experiments to test the feasibility of an mRNA-based screen and to assess whether changes known to happen at the protein level are also detectable at the mRNA level. We expected changes at RNA level to happen faster than at protein level. Based on the results obtained in the previous section, the 1:1 (CTL:melanoma cell) ratio and the 24-h time-point were chosen for mRNA analysis to obtain sufficient numbers of surviving tumor cells. Indeed, in a preliminary experiment, the T1185B melanoma cell line yielded sufficient mRNA after co-culture or cytokine treatment to perform qPCR experiments. In accordance with the protein expression, HLA Class I mRNA was upregulated in the melanoma cell line after treatment with MelanA-specific CTLs or cytokines but not after treatment with non-specific CTLs (data not shown). MelanA mRNA decreased under these conditions in line with the observation that MelanA protein was downregulated after treatment with MelanA-specific CTLs or cytokines but not non-specific CTLs (Figure S6 in Supplementary Material) and in agreement with the literature (26). Our observations show that the changes at protein level can also be detected at the mRNA level with the 1:1 ratio and the 24-h time-point.

**Figure 5:**
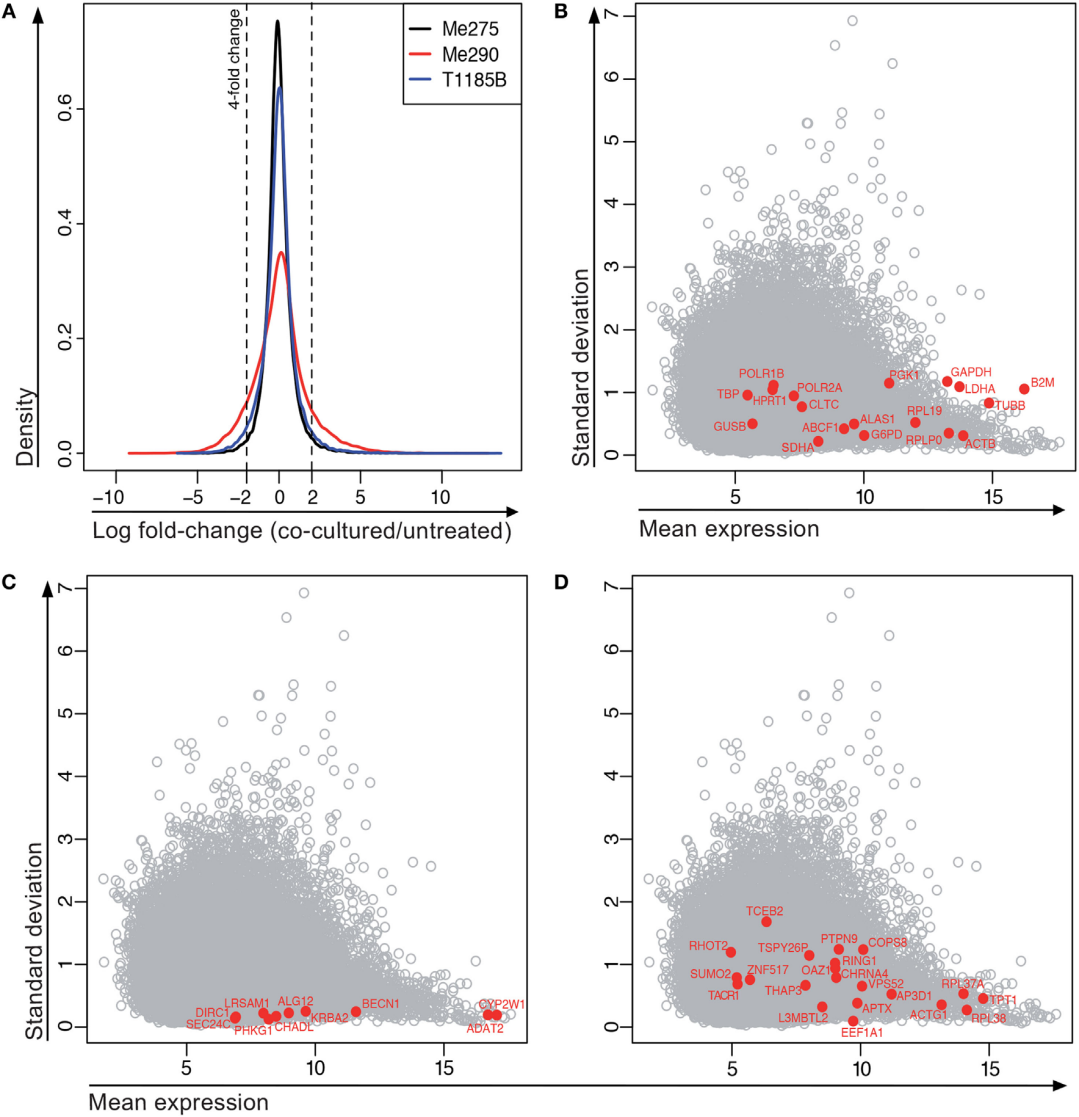
**Gene expression of co-cultured compared to untreated melanoma cell lines**. **(A)** Distribution of all log fold-changes between the treated and untreated condition for each of the cell lines. *N* = 41,839 probes, bandwidth = 0.06145. **(B–D)** Expression of “housekeeping” genes in microarray data set. **(B)** Frequently used normalization genes. **(C)** Stably expressed genes with <0.25 SD across all samples and stable expression according to the Genevestigator tool. **(D)** Control genes suggested by the RefGenes tool in Genevestigator.

Next, the microarray-based screen was performed on four melanoma cell lines, comparing 24-h treatment with MelanA-specific CTLs versus untreated. Following culture, 1000 melanoma cells were isolated from the co-culture by FACS (Figure  S7 in Supplementary Material) and relative RNA expression was measured using a gene expression microarray [Agilent Whole Human Genome 8 × 60K (V2)]. The co-culture of the fourth cell line, T1015A, had to be omitted from the analysis due to insufficient sample quality (Figure S8 in Supplementary Material), illustrating that isolation of cells with sufficiently high mRNA quality from the co-culture is a technical challenge. From analysis of the three qualified co-cultures, 212 genes were at least fourfold differentially expressed between co-cultured and untreated samples across all three melanoma cell lines (184 genes increased and 28 decreased; Figure 5A). Thus, our results show that mRNA screening of the co-culture system is technically feasible.

We wanted to confirm the reproducibility of the microarray results using a second frequently used mRNA-based technology. We chose the NanoString technology because it is highly quantitative and sensitive allowing the detection of several hundred individual mRNAs with a high dynamic range in a single reaction containing as little as 100 ng RNA. Furthermore, the technology is based on hybridization and does not require additional amplification or enzymatic steps making it less susceptible to unwanted bias (27). Finally, it allows studying more experimental conditions at a more advantageous cost, as compared to microarray techniques.

### Identification of Appropriate Normalization Genes for NanoString Analyses: *ALG12, GUSB, RPLP0, KRBA2* and *ADAT2* Are Stably Expressed Genes and Not Influenced by CTLs or Their Cytokines

For differential gene expression analysis by NanoString, a set of stably expressed genes is required to normalize for potential differences in loaded RNA quantities between samples. This is necessary since “global” normalization methods, such as those typically used for, e.g. microarray and RNA-seq data, are not appropriate in situations where only relatively few, non-randomly chosen genes are studied. However, the expression of some of the frequently used housekeeping genes in the above-mentioned microarray, including *GAPDH, B2M* and *HPRT1*, showed a large variability in expression among our samples (Figure 5B). Indeed, *B2M* expression is regulated by IFNγ, a cytokine secreted by activated CTLs (28). *GAPDH* is involved in functional networks that might be deregulated in cancer cells and is not suitable for studies in cancer cell lines (29). *HPRT1* was expressed less stably than other housekeeping genes in a publication evaluating housekeeping genes for the study of heart disease (30). The authors found *HPRT* expression to be influenced by age and hypoxia. Consequently, it is critical for the interpretation of gene expression data to confirm the stable gene expression of frequently used normalization genes under co-culture conditions and to identify additional suitable normalization genes if necessary.

To find a collection of stably expressed genes that can be used to normalize gene expression from the very high to very low expression range, we first extracted all genes with a SD below 0.25 in the microarray experiment and the average expression level for these genes (average expression across all samples and cell lines). From this list, we evaluated some of the genes with Genevestigator,^1^ to see the variability of their expression across a large number of microarray experiments. From this evaluation, the most promising candidates are visualized in Figure 5C. Among these genes, at least *BECN1, SEC24C*, and *DIRC1* also showed stable expression based on GeneProf,^2^ which is based on RNA-seq data. We did a search for stable genes using the RefGenes tool in Genevestigator, and found new candidates, but again only a fraction of these candidates were stably expressed in the microarray (Figure 5D). Based on these analyses, we chose eight genes for normalization of future mRNA analysis with the NanoString platform (*ADAT2, ALG12, DIRC1, GUSB, KRBA2, PHKG1, RPLP0*, and *SLC26A3*).

**Figure 6:**
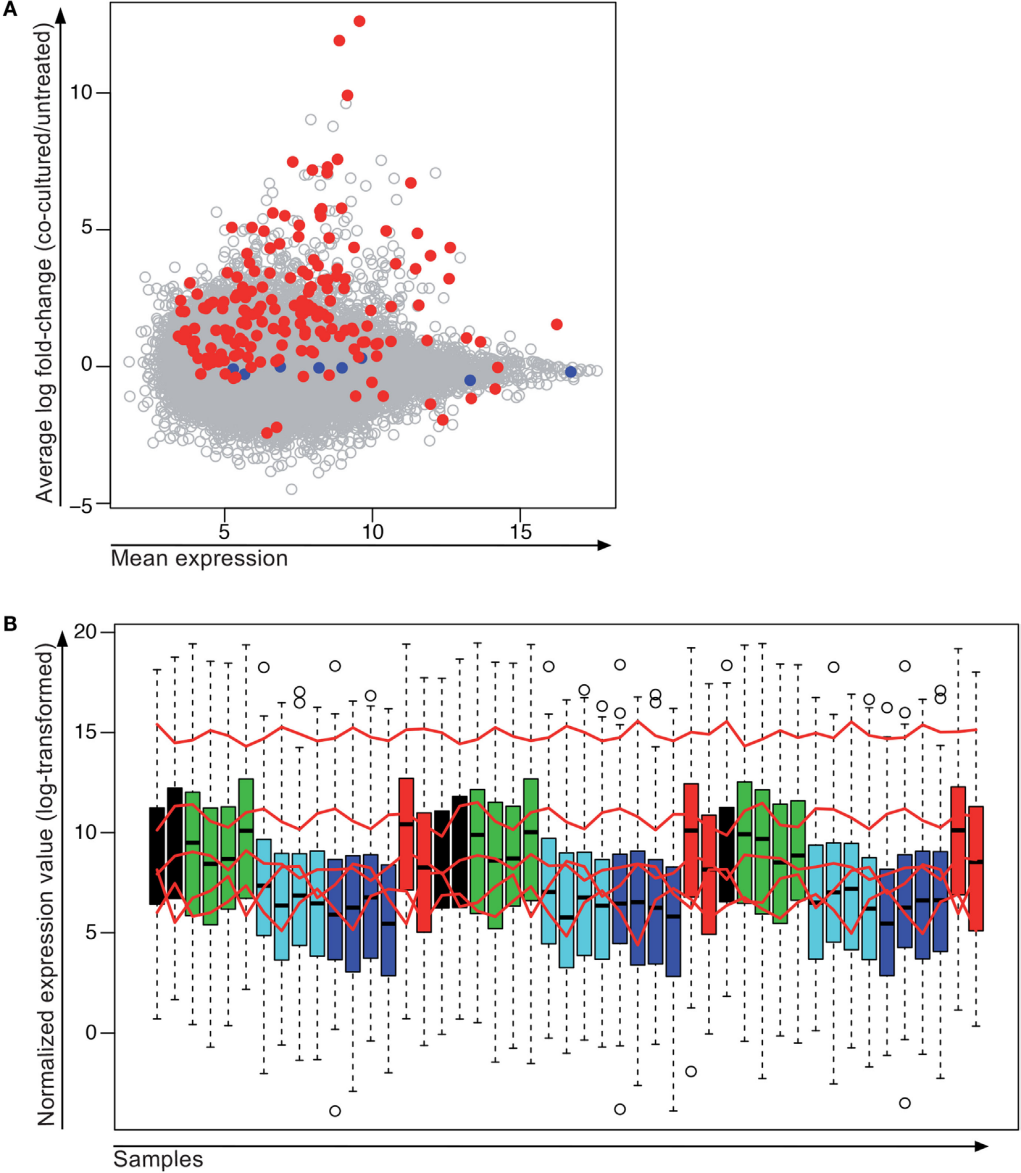
**Stably expressed genes detected by microarray and NanoString**. **(A)** 185 genes with different expression levels were chosen for quantification by NanoString. Shown is the average expression measured by microarray from all samples on the *x*-axis and the average log fold-change between the co-cultured and the non-co-cultured samples across the three melanoma cell lines (Me290, Me275, and T1185B) on the *y*-axis. For the planned NanoString, the normalization genes (blue) were chosen to span a wide range of expression values but without changes between the treated and untreated cell lines. Red dots, genes selected for NanoString; blue dots, normalization genes for NanoString; gray dots, genes that were only measured by microarray. **(B)** Normalized gene expression values of all samples analyzed by NanoString. Red lines indicate expression levels of genes used for normalization (order from highest to lowest expression: *RPLP0, GUSB, ALG12, ADAT2* and *KRBA2*). Treatment color code: Black, MelanA-specific clone 1; green, cytokines; cyan, negative control CTLs; blue, untreated; red, MelanA-specific Clone 121. Samples are shown in the same order as they were run on the NanoString cartridge.

We then assembled a set of 185 genes covering all expression levels and degrees of differential gene expression in the microarray to study their expression by NanoString (Figure 6A). For this experiment, we pooled the cells of several 24-wells containing the same treatment condition in order to collect higher numbers of living melanoma cells (between 3 and 12 wells depending on the culture condition and the number of viable cells expected with a given treatment). Out of the eight chosen control genes, three were not used for normalization because they showed a very low level of expression (*DIRC1, SLC26A3*, and *PHKG1*). The remaining control genes (*ALG12, GUSB, RPLP0, KRBA2*, and *ADAT2*) that showed sufficiently high gene expression were used for normalization (Figure 6B).

In addition to the purity check upon cell sorting, potential CTL contamination in melanoma cell samples isolated from the co-cultures was checked by including a series of CTL markers on the NanoString (*CD3D, CD3E, CD3G, CD8A* and *CD8B*). In all samples, the normalized expression values of all CTL markers were below the threshold for background, except for *CD3D*, for which two culture conditions (Me275 and T1185B with MelanA-specific Clone 1) were slightly above the threshold but still very low. Since the *CD3* and *CD8* levels were also slightly increased in the cytokine-treated samples (i.e. in absence of CTLs), we assume that the potential CTL contamination was negligible, that the probe showed cross reactivity or that these genes might be expressed at low background levels by the melanoma cell lines (Figure S9 in Supplementary Material).

**Figure 7:**
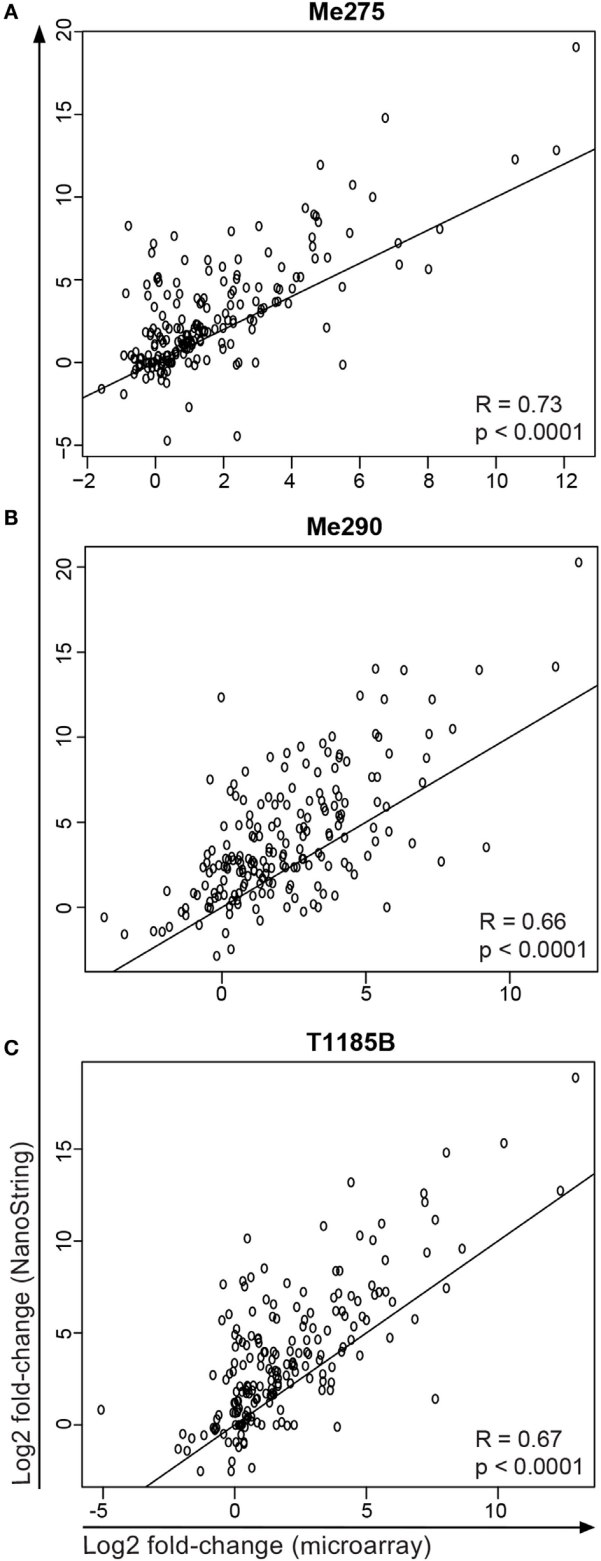
**Comparison of microarray and NanoString data for samples and genes that were measured in both experiments**. Shown is the log2 fold-change (co-cultured/untreated) of the three melanoma cell lines **(A)** Me275, **(B)** Me290 and **(C)** T1185B.

Finally, we compared the results of the microarray and of the NanoString experiments. The differential gene expression in the microarray correlated well with the NanoString, but the microarray generally underestimated the log fold-change compared to the NanoString (Figure 7). The reason for this may be that the background intensities on the microarray may lead to more heavily biased fold-changes compared to the NanoString. It has previously been observed that microarray analysis show global compression that is most extreme for very high and low gene expression values (31). In addition, for the NanoString, more cells were sorted (30,000–300,000 cells per sample for the NanoString experiment versus 1000 cells per sample in the microarray experiment) and no global amplification of the isolated mRNA was necessary. Together, our data show that sufficient and high-quality mRNA can be isolated from the co-culture system to perform genome-wide differential gene expression analysis and that the experimental system yields reproducible results.

## Discussion

It is becoming increasingly clear that CTLs can not only kill cancer cells but also provoke changes of the tumor microenvironment with consequences that may impact on the patient’s clinical outcome. Here, we describe the setup of an *in vitro* co-culture system of human melanoma cell lines and melanoma-specific CTLs, including quality control and fine-tuning of experimental parameters. Previously published approaches describe mouse models and long-term culture escape variants of tumors and cell lines (4, 6–8, 32). By contrast, our two-component *in vitro* system allows to study the CTL–tumor cell interactions in human material and the analysis of much more rapidly occuring reactions in a widespread and detailed manner using well-defined and reproducible culture conditions.

The tumor microenvironment is infiltrated with different types of immune and other host cells. In this heterogeneous tissue, it is difficult to study cause–effect relationships of specific cell types. The two-component-culture system described here is suitable to study the CTL–melanoma cell relationship because no other cell type or stroma component is confounding the interaction. This is a reductionist model that excludes interactions with other cell types of the tumor microenvironment, thus conversely, the model alone does not allow direct conclusions on *in vivo* functions.

With this co-culture setup, we could successfully isolate sufficient numbers of viable cells for protein and mRNA analyses with high-quality data, including genome-wide screens and broad quantitative gene expression analysis. Importantly, our mRNA-based microarray and NanoString screens revealed that frequently used normalization genes are not stably expressed in melanoma cells exposed to melanoma-specific CTLs or their cytokines (IFNγ/TNFα). In turn, we identify *ALG12, GUSB, RPLP0, KRBA2* and *ADAT2* to be suitable normalization genes for expression analysis in our co-culture system. Beyond the culture system described here, these normalization genes could find wide use in studies of immune-related culture and expression systems and even in direct *ex vivo* analysis of human surgery specimens in which CTLs or other cytokine-secreting cells are present.

In addition to studying direct gene expression changes of melanoma cells upon exposure to CTLs, this co-culture system can be employed for a series of yet unanswered questions. As an example, assessing the reversibility of rapid reactions in the co-cultured melanoma cell lines may be important for the understanding of CTL–melanoma cell dynamics. In a study by Landsberg et al. melanoma-bearing mice relapsed after adoptive T cell therapy. Interestingly, the relapsing tumors showed inflammation-induced reversible changes of melanocytic antigen expression. The authors proposed a dynamic equilibrium between differentiated and dedifferentiated tumor cells (8). Our culture system is suitable to address whether this equilibrium or other CTL-induced changes can also be observed *in vitro*. To study reversibility, we re-cultured melanoma cells that were isolated from a 24-h co-culture with CTLs. One out of four tumor cell lines grew back to sufficient density for analysis, demonstrating the potential of our co-culture system to answer this question. In this preliminary analysis, we found that the expression of the target antigen MelanA was lost in half of the living tumor cells, but returned to normal levels in cultures (without CTLs) after further 6 and 16 days, suggesting that decreased expression of MelanA is reversible. This finding is in line with a previously published study of T cell therapy in a mouse melanoma model (8). Similarly, the inhibitory receptor ligand PDL1 is upregulated at RNA and protein level in MelanA-specific co-cultures, but decreased to baseline levels 6 and 16 days after melanoma cells were isolated from the co-culture. However, more experiments are necessary for definitive conclusions.

Another example is based on the observation that the peptide repertoire on the surface of tumor cells can be shaped by CTLs. It has been reported that CTLs with low affinity for their target antigen may strip target-peptide-loaded major histocompatibility complexes (pMHC) from the surface of tumor cells without lysing them (33). Subsequently, the lower abundance of target antigen on the tumor cell surface may be insufficient for lysis, even by high-affinity CTLs. However, this study did not analyze whether additional changes occurred in the tumor cells. In our co-culture system, the melanoma-specific CTLs have been chosen based on their ability to lyse melanoma cells. It would be interesting to investigate the effects of melanoma-specific low-affinity CTLs that lack the ability to lyse their target cell in our experimental system.

## Conclusion

Our short-term co-culture system is well suited to characterize the early and dynamic interplay of melanoma cells and CTLs because the setup allowed the isolation of sufficient viable cancer cells for high-quality protein and mRNA expression data. We furthermore, identified appropriate genes for normalization of gene expression data that are not the commonly used as standard normalization genes. Our system has the potential to address questions relevant for T cell-based immunotherapies that are directly related to the effects of CTL attack on cancer cells.

## Author Contributions

Conceived and designed the experiments: NN, CS, PB, DR, SFM, MD, and DS. Performed the experiments: NN and SFM. Analyzed the data: NN, CS, DB, and SFM. Wrote the paper: NN, CS, DB, PB, DR, MD, SFM, and DS.

## Conflict of Interest Statement

The authors declare that the research was conducted in the absence of any commercial or financial relationships that could be construed as a potential conflict of interest.

## Footnotes

^1^www.genevestigator.com

^2^www.geneprof.org
