# Supplementary material for: A Well-Controlled Experimental System to Study Interactions of Cytotoxic T Lymphocytes with Tumor Cells
Source: Front Immunol. 2016 Aug 30;7:326. doi: 10.3389/fimmu.2016.00326 (PMC5003846; doi:10.3389/fimmu.2016.00326)
Supplement: Supplementary file 1 [file Data_Sheet_1.DOCX]

Supplementary Material

An Experimental System To Study Interactions Of Cytotoxic T Lymphocytes With Tumor Cells

Natalie J. Neubert^1^, Charlotte Soneson^2,3^, David Barras^2^, Petra Baumgaertner^1^, Donata Rimoldi^1^, Mauro Delorenzi^2,4^, Silvia A. Fuertes Marraco^1^ and Daniel E. Speiser^1^*

*** Correspondence:**Daniel Speiser, Clinical Tumor Biology & Immunotherapy Group, Department of Oncology, Ludwig Cancer Research of the University of Lausanne, Biopole 3 - 02DB92, Ch. des Boveresses 155, 1066 Epalinges, Switzerland. Phone: 41-21-314-01-82; Fax: 41-21-692-59-95; E-mail: doc@dspeiser.ch

# Supplementary Figures

Supplementary Figure S1. Antigen-specific co-culture: Melanoma cell lines express the target antigen MelanA, while CTLs express MelanA-specific TCRs. (A) All melanoma cell lines chosen for co-cultures were MelanA and HLA Class I positive as revealed by flow cytometry analysis. Specifically stained melanoma cell lines are indicated in black and isotype-matched controls in grey. Red numbers show gate frequency of stained samples. (B) MelanA-specific CTLs (Clone 121 and Clone 1), but not Epstein-Barr-virus-specific CTLs (Clone 12) stained positively with MelanA-A2 tetramers, indicating that they expressed MelanA-specific TCRs on their surface. All CTLs were CD8 positive. Black dots show stained CTLs and grey dots show unstained CTLs.

Supplementary Figure S2. MelanA-specific CTLs kill MelanA-positive but not MelanA-negative melanoma cells, whereas yellow-fever-virus (YFV)-specific CTLs do not kill melanoma cell lines. The killing capacity of CTLs was measured in a 4h ^51^Cr release assay. (A) MelanA-positive melanoma cell lines with MelanA-specific CTLs. The MelanA peptide ELAGIGILTV was added as positive control. (B) MelanA-negative melanoma cell line (Na8) with MelanA-specific CTLs showing no killing in absence of synthetic MelanA peptide ELAGIGILTV. (C) YFV-specific CTL clone N8 showed no killing of MelanA-positive melanoma cell lines in absence of synthetic YFV peptide LLWNGPMAV. N = 2, error bars indicate standard deviation.

Supplementary Figure S3. Gating strategy for flow cytometric analysis of co-cultured cells. (A) Exclusion of debris in the forward-sideward scatter plot. CTLs and melanoma cells are separated based on CFSE and violet tracker expression. For each cell population, doublets are excluded followed by isolation of living (Vivid-negative) cells. Shown is one representative co-culture of Me275 melanoma cells with MelanA-specific Clone 1 after 24h co-culture. (B) Morphology changes during co-culture make it difficult to separate CTLs from melanoma cells based on forward and sideward scatter. Shown is one representative co-culture of Me290 melanoma cells with MelanA-specific Clone 121.


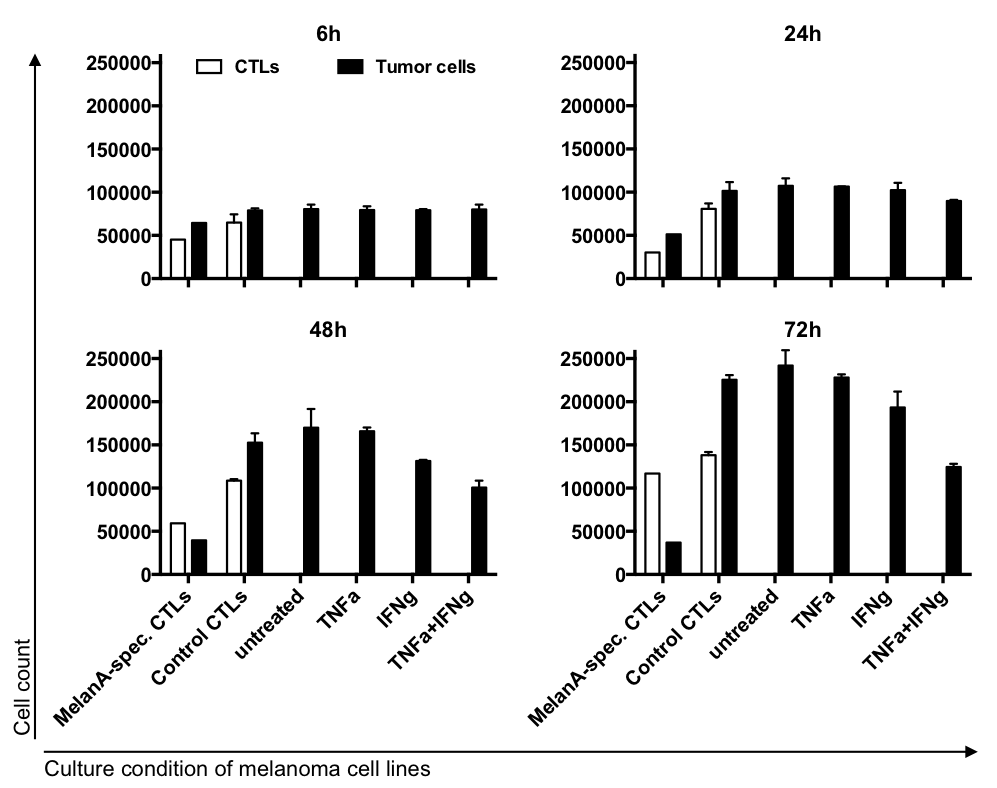


Supplementary Figure S4. Counts of living cells of a representative co-culture with the melanoma cell line Me275. Co-cultures were seeded at a 1:1 ratio of CTLs with melanoma cells. N=2, except the MelanA-specific CTL condition N = 1. Shown is mean ± standard deviation.


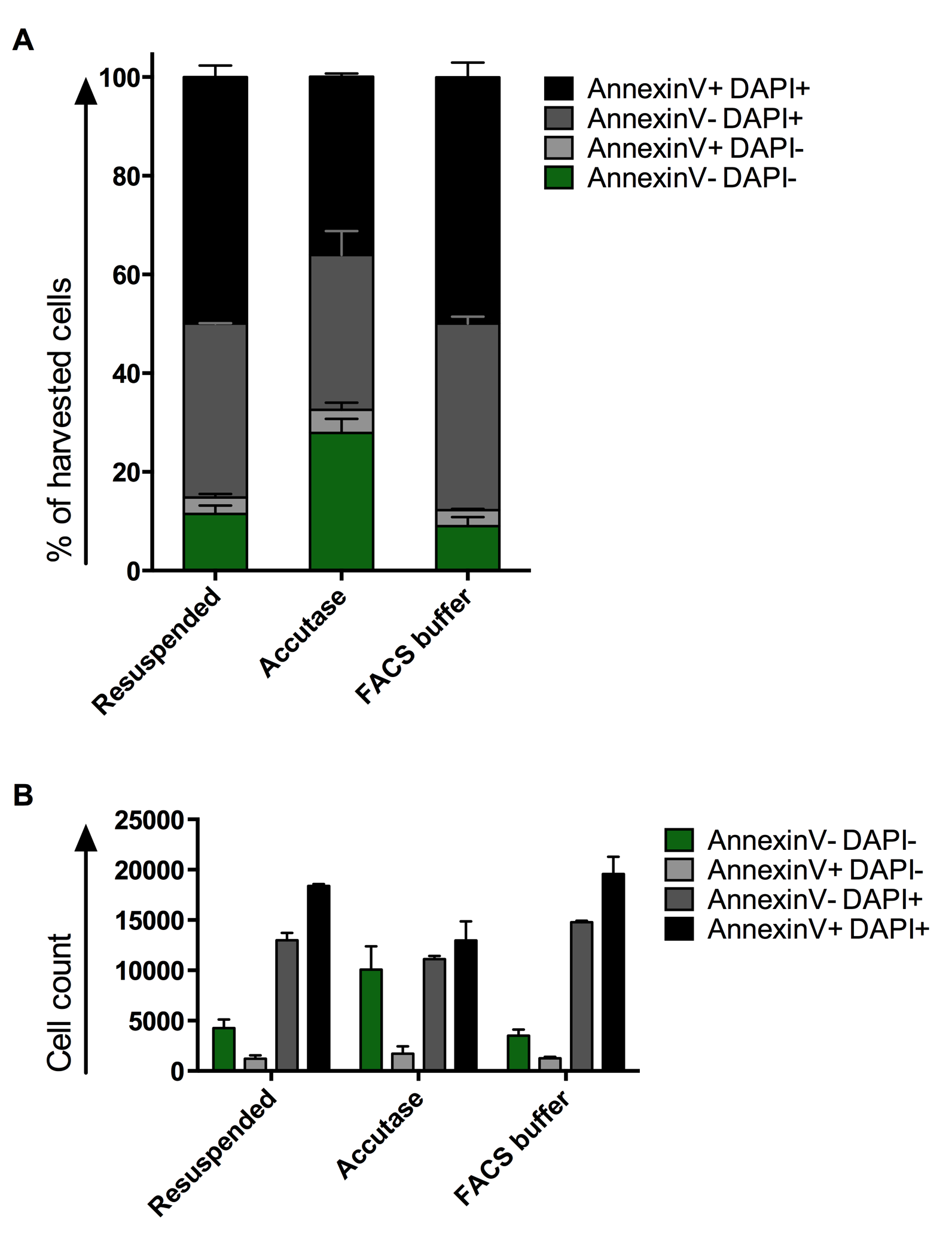


Supplementary Figure S5. Viability of melanoma cells using different harvesting methods. Resuspended: cells were detached by repeated pipetting of the medium. Accutase: supernatant was removed, the 24-wells were washed once with PBS, cells were briefly incubated with 100µl accutase at 37°C, and detached cells were collected with 500 µl fresh medium. FACS buffer: supernatant was removed, 1 ml FACS buffer was added (PBS supplemented with 5 mM EDTA, 0.2% BSA and 0.2% NaAzide), cells were left 5-10 min on ice and then detached cells were collected. (A) Percent of total, (B) absolute cell counts. Shown is the mean ± standard deviation. N = 2.

Supplementary Figure S6. MelanA-specific CTLs (green) or CTL-derived cytokines (black) but not non-specific CTLs (blue) decreased MelanA protein expression in the melanoma cell line T1185B. Untreated cells are shown in red. Measurements were performed using a flow cytometer. N = 2, error bars indicate standard deviation.


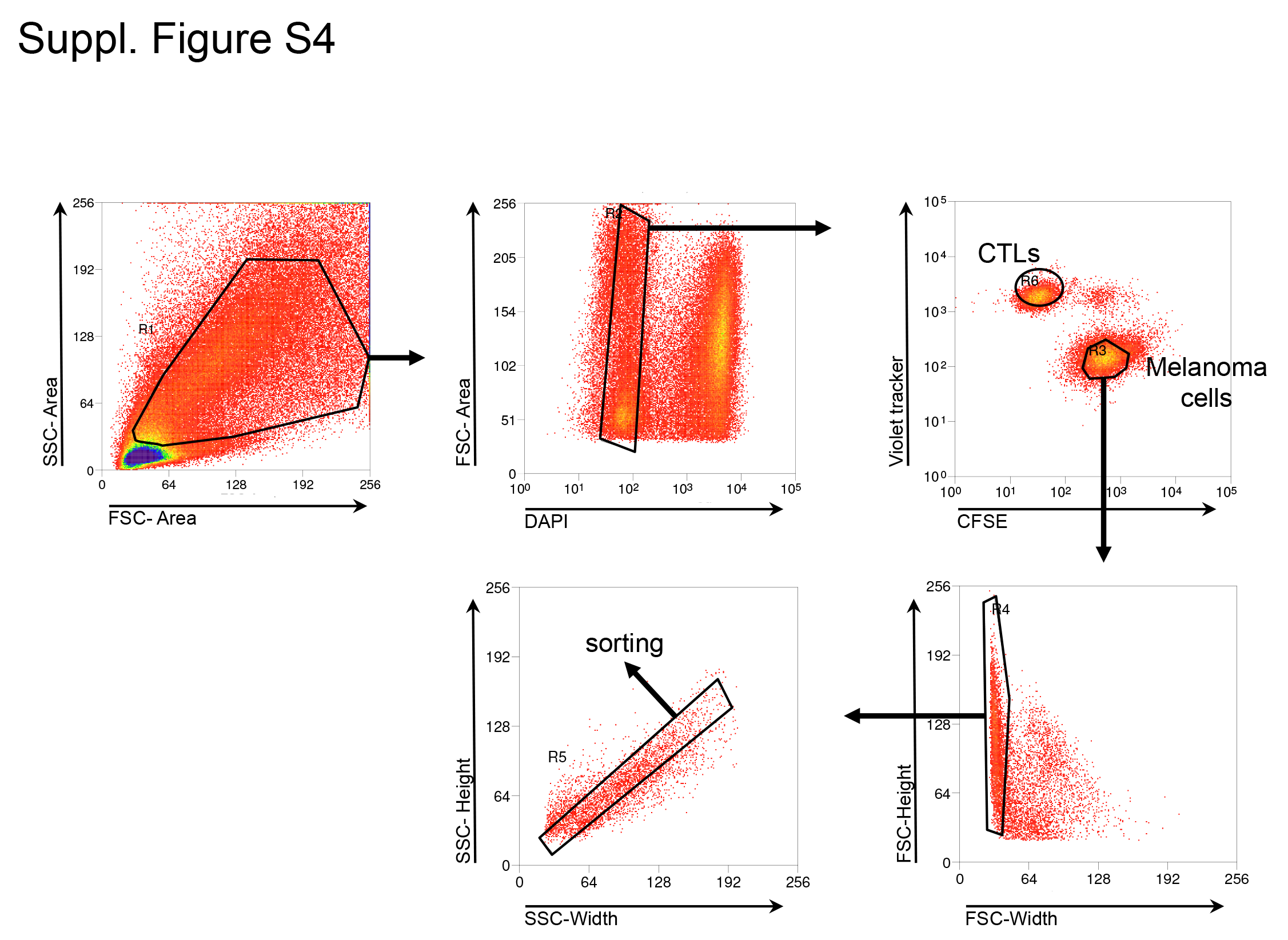


Supplementary Figure S7. Sorting strategy. Living melanoma cells were isolated by gating on DAPI negative CFSE positive cells followed by doublet exclusion.

Supplementary Figure S8. Quality of samples used for microarray analysis. (A) Gel image and (B) electropherogram of amplified cDNA samples. As a reference, the DNA molecular weight ladder (in nucleotides, nt) is shown in the first lane. The lowest migrating, green band represents an internal standard. Scaling of the y-axis is done automatically, relative to the strongest signal within a single run.


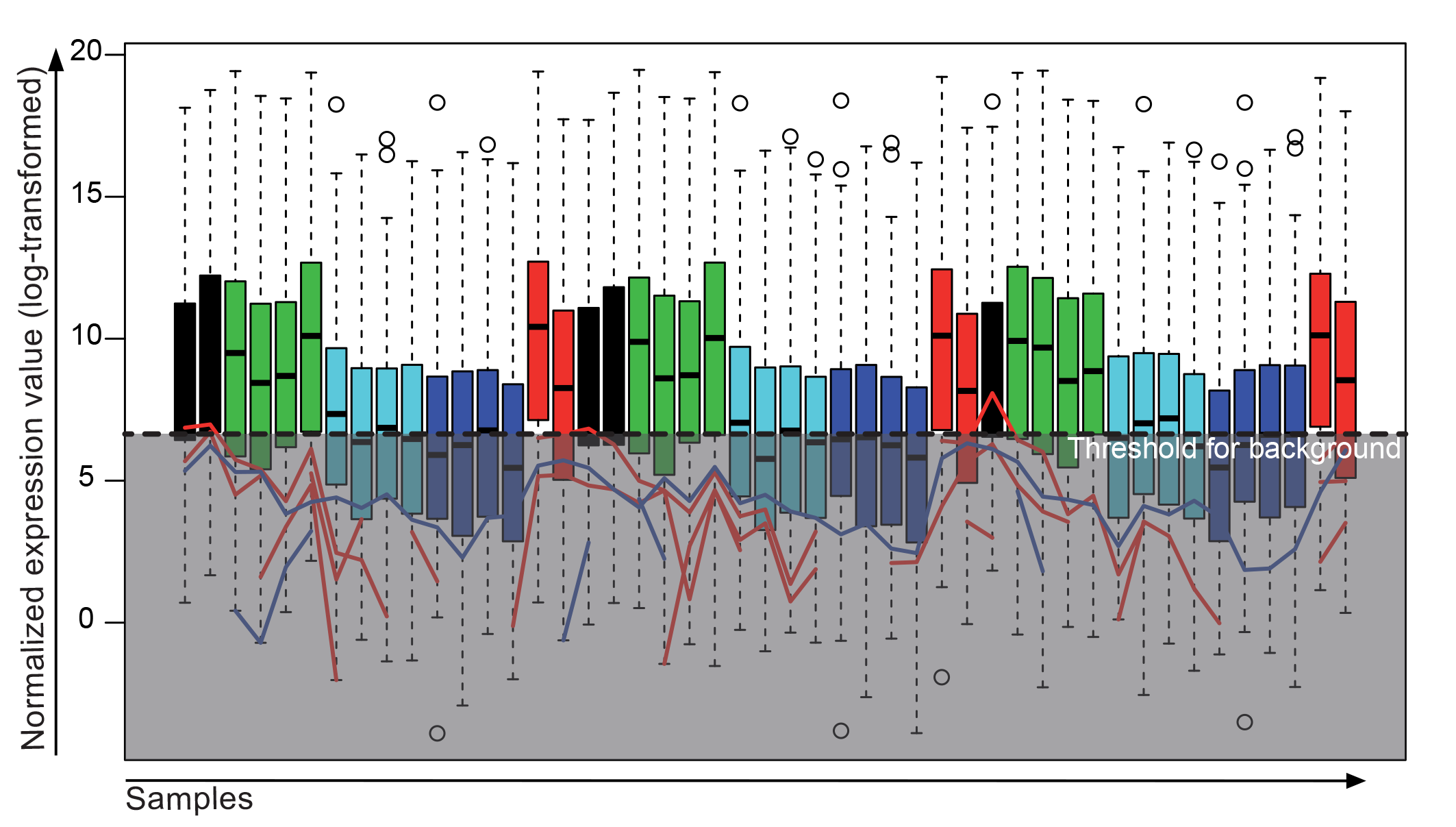


Supplementary Figure S9. Normalized gene expression values of all melanoma cell line samples analyzed by NanoString. Red lines indicate expression levels of *CD3* (*CD3D, CD3E, CD3G*) and blue lines expression levels of *CD8* (*CD8A* and *CD8B*). Treatments are indicated with color codes (in the order appearing on the graph): Black, MelanA-specific clone 1; green, cytokines (IFNγ and TNFα); cyan, negative control CTLs; blue, untreated; red, MelanA-specific Clone 121. Samples are shown in the same order as they were run on the NanoString cartridge. For each cell line and culture condition three replicates were measured except for T1185B with clone 1 for which two replicates were measured.
